# Fungicide ingestion reduces net energy gain and microbiome diversity of the solitary mason bee

**DOI:** 10.1038/s41598-024-53935-y

**Published:** 2024-02-08

**Authors:** Mitzy F. Porras, Juan Antonio Raygoza Garay, Malachi Brought, Tomas López–Londoño, Alexander Chautá, Makaylee Crone, Edwin G. Rajotte, Ngoc Phan, Neelendra K. Joshi, Kari Peter, David Biddinger

**Affiliations:** 1 Department of Entomology, The Pennsylvania State University, 501 ASI Bldg, University Park, USA; 2 Department of Communication Sciences and Disorders, Holden Comprehensive Cancer Center, University of Iowa, 200 Hawkins Dr, Iowa City, IA 52242 USA; 3 Department of Biology, The Pennsylvania State University, 208 Mueller Lab, University Park, PA16802 USA; 4 Department of Ecology, Cornell University, Ithaca, NY 14850 USA; 5 Center for Pollinator Research, Intercollege Graduate Program in Ecology, Huck Institutes of the Life Sciences, Pennsylvania State University, University Park, PA16802 USA; 6 Department of Entomology and Plant Pathology, University of Arkansas, Fayetteville, AR 72701 USA; 7 Department of Plant Pathology and Environmental Microbiology, Fruit Research and Extension Center, Pennsylvania State University, 290 University Dr., Biglerville, PA 17307 USA; 8 Department of Entomology, Fruit Research and Extension Center, 290 University Dr., Biglerville, PA 17307 USA; 9 Present Address: Department of Biology, San Francisco State University, 1600 Holloway Avenue, San Francisco, CA 94132 USA

**Keywords:** Fungicide risk assessment, Host-microbial diversity, *Osmia cornifrons*, Pollen provisions, Mortality, Environmental impact, Applied microbiology, Agroecology

## Abstract

Fungicides are frequently used during tree fruit bloom and can threaten insect pollinators. However, little is known about how non-honey bee pollinators such as the solitary bee, *Osmia cornifrons,* respond to contact and systemic fungicides commonly used in apple production during bloom. This knowledge gap limits regulatory decisions that determine safe concentrations and timing for fungicide spraying. We evaluated the effects of two contact fungicides (captan and mancozeb) and four translaminar/plant systemic fungicides (cyprodinil, myclobutanil, penthiopyrad, and trifloxystrobin) on larval weight gain, survival, sex ratio, and bacterial diversity. This assessment was carried out using chronic oral ingestion bioassays where pollen provisions were treated with three doses based on the currently recommended field use dose (1X), half dose (0.5X), and low dose (0.1X). Mancozeb and penthiopyrad significantly reduced larval weight and survival at all doses. We then sequenced the 16S gene to characterize the larvae bacteriome of mancozeb, the fungicide that caused the highest mortality. We found that larvae fed on mancozeb-treated pollen carried significantly lower bacterial diversity and abundance. Our laboratory results suggest that some of these fungicides can be particularly harmful to the health of *O. cornifrons* when sprayed during bloom. This information is relevant for future management decisions about the sustainable use of fruit tree crop protection products and informing regulatory processes that aim to protect pollinators.

## Introduction

The solitary mason bee *Osmia cornifrons* (Hymenoptera: Megachilidae) was introduced to the United States from Japan in late 1970s and early 1980s^1^, ever since then, the species has played an essential role as a pollinator in managed ecosystems. Naturalized populations of this bee are part of the approximately 50 wild species of bees that have supplemented the honey bee for pollination of almond and apple orchards in the USA^2,3^. Mason bees face numerous challenges, including habitat fragmentation, pathogens, and pesticides^3,4^. Among pesticides, fungicides can reduce energy gain, foraging^5^ and fitness^6,7^. Although recent studies indicate mason bee fitness is directly affected by symbiotic and exobiotic microbes^8,9^ because bacteria and fungi can influence nutrition and immune response, the effects of exposure to fungicides on microbial diversity of mason bees are just beginning to be explored.

Before and during flowering, tree fruit orchards are sprayed with fungicides with varying modes of action (contact and systemic) to treat diseases such as apple scab, bitter rot, brown rot, and powdery mildew^10,11^. Fungicides were assumed harmless to pollinators; hence, they were recommended to growers during flowering. Contact and ingestion exposure of these fungicides to honey bees are relatively well known since it is part of the pesticide registration process by the U. S. Environmental Protection Agency and many other countries' regulatory agencies^12–14^. However, the effects of fungicides on non-honey bees are less well known as they are not required as part of the registration protocols in the US^15^. In addition, there is a general lack of standardized testing protocols for solitary bees^16,17^, and it is challenging to maintain colonies that provide bees for testing^18^. Various species of managed *Osmia* in Europe and the US are increasingly being tested to examine pesticide effects on wild bees, and recently, a standardized protocol was developed for *O. cornifrons*^19^.

*Osmia cornifrons* is univoltine and has been commercially used in tree fruit crops to supplement or replace honey bees. These bees emerge between March and April, with protandrous males emerging three to four days ahead of females. After mating, females actively collect pollen and nectar for provisioning a series of brood cells within tubular nest cavities (natural or artificial)^1,20^. Eggs are laid on the pollen provisions within a cell; the female then builds a mud partition before provisioning the next cell^21^. The first larval instar is enclosed within the chorion, feeding on embryonic fluids. From the second to fifth instar (prepupa), the larva feeds on pollen provisions^22^. Once the pollen provision is completely consumed, the larva forms a cocoon, pupates and becomes an adult in the same brood cell, usually by the end of summer^20,23^. The adult emerges in the following spring^22^. Adult survival is correlated with net energy gain (weight gain) based on the provisions consumed. Therefore, the nutritional quality of the pollen, as well as other factors such as weather or pesticide exposure, are determinants of survival and fitness^24^.

Pre-bloom applications of insecticides and fungicides with the ability to move within the plant vascular system to varying degrees from translaminar (e.g., able to move from the top surface of a leaf to the bottom surface as with some fungicides)^25^ to genuinely systemic neonicotinoid insecticides that can move from root applications up into the canopy have been previously shown to move into the nectar of apple bloom^26^ where they can kill adult *O. cornifrons*^27^. Some pesticides can also move into the pollen, affecting *O. cornifrons* larval development and causing mortality^19^. Other studies have shown that some fungicides can dramatically change nesting behavior in a congener, *O. lignaria*^28^. Furthermore, laboratory and field studies simulating pesticide (including fungicides) exposure scenarios demonstrated adverse effects on physiology^22^, morphology^29^, and survival in honey bees and some solitary bees^12^. The impact of various fungicide sprays applied directly to open flowers during bloom, which would contaminate the pollen collected by adult *O. cornifrons* for larval development, has yet to be explored^30^.

It is increasingly recognized that larval development is affected by the microbial community present in the pollen and digestive system. The bee microbiome influences parameters such as body mass^31^, metabolism alterations^22^, and susceptibility to pathogens^32^. Prior research has investigated the effects of developmental stages, nutrients, and environment on solitary bee microbiome. These studies revealed similarities in structure and abundance of the microbiome of both larvae and pollen^33^ and the most abundant bacteria genera, *Pseudomonas* and *Delftia*, in solitary bee species^34,35^. However, the impact of fungicides on the larval microbiome through direct oral exposure remains unexplored despite its relevance for strategies aimed at preserving bee health.

This study tested the effects of field-realistic doses of six commonly used fungicides registered for use on tree fruit throughout the US, which included contact and systemic fungicides via oral exposure to *O. cornifrons* larvae from contaminated provisions. We found contact and systemic fungicides reduced bee weight gain and increased mortality, with the most severe impact associated with mancozeb and penthiopyrad. Then, we compared the microbial diversity of larvae fed with pollen provisions treated with mancozeb against those fed with control provisions. We discuss potential mechanisms underpinning the lethality as well as implications for integrated pest and pollinator management (IPPM) programs^36^.

## Material and methods

### Rearing method and egg collection

Overwintering adults of *O. cornifrons* in cocoons were obtained from the Fruit Research Center in Biglerville, PA and stored at **− **3 to 2 °C (± 0.3 °C). before the experiment (a total of 600 cocoons). In May 2022, groups of 100 cocoons of *O. cornifrons* were transferred daily to plastic cups (50 cocoons/cup, 5 cm DI × 15 cm length) with a napkin introduced inside the cup to aid emergence and provide a substrate for chewing, which reduces mason bee stress^37^. Two plastic cups with cocoons were placed inside an insect cage (30 × 30 × 30 cm, BugDorm MegaView Science Co. Ltd., Taiwan) with a 10 ml feeder containing 50% sucrose solution and held for four days to ensure emergence and mating at 23 °C with 60% RH and photoperiod 10 L (low intensity): 14 D. One hundred individual mated females, and males were released every morning for six days at the peak of apple bloom (100 individuals/day) in two artificial nest boxes (trap nests: width 33.66 × height 30.48 × length 46.99 cm; Supplementary Fig. 1) placed at The Arboretum at Pennsylvania State University, PA near cherry (*Prunus cerasus* 'Eubank' Sweet Cherry Pie™), peach (*Prunus persica* 'Contender,' *Prunus persica* 'PF 27A' Flamin Fury^®^), pear (*Pyrus pyrifolia* 'Olympic,' *Pyrus pyrifolia* 'Shinko,' *Pyrus pyrifolia* 'Shinseiki'), sweet crabapple (*Malus coronaria*) and numerous apple tree varieties (*Malus coronaria*, *Malus domestica* 'Co-op 30' Enterprise™, *Malus domestica* 'Co-Op 31' Winecrisp™, *Malus domestica* 'Freedom,' *Malus domestica* 'Golden Delicious', *Malus domestica* 'Nova Spy'). Each blue plastic nest box was placed on top of two wooden crates**.** Each nest box contained 800 empty Kraft tubes (spirally wound open-ended 0.8 cm inside diameter × 15 cm length) (Jonesville Paper Tube Corp, MI) with an inserted opaque glassine tube (0.7 cm outside diameter × 15 cm length) with plastic plugs (T-1X Caplugs) to provide nest sites.

Both nest boxes were faced east, covered with a green plastic garden fence (Everbilt Model# 889250EB12, hole size 5 × 5 cm, 0.95 m × 100 m) to keep rodents and birds out, and provided with mud on the soil surface near the nest boxes (Supplementary Fig. 1a). *Osmia cornifrons* eggs were harvested daily from the nest boxes by collecting 30 tubes and bringing them to the laboratory. An incision was made on the end of the tube using scissors; then, we unraveled the spiral tubing, revealing the brood cells. Individual eggs and their pollen provisions were removed using a bent spatula (Microslide Tool Set BioQuip Products Inc., CA). The eggs were incubated on moist filter paper and placed in Petri dishes for 2 hours^38^ and then used in our experiments (Supplementary Fig. 1b–d).

### Fungicide exposure

In laboratory we evaluated the oral toxicity of six fungicides applied before and during apple bloom, at three concentrations (0.1X, 0.5X, and 1X, where 1X is the labeled high field dose applied in 100 gal water/acre = field concentration, Table 1). Each concentration was replicated 16 times (*n* = 16). Toxicity of two contact fungicides (Table S1: mancozeb at 2696.14 ppm and captan at 2875.88 ppm) and four systemic fungicides (Table S1: Penthiopyrad at 250.14 ppm; Trifloxystrobin at 110.06 ppm; Myclobutanil at 75.12 ppm; Cyprodinil at 280.845 ppm) which are widely used in fruits, vegetables, and ornamental crops. We homogenated pollen provisions using a grinder and transferred 0.20 g into a well (24–-Well Falcon Plate), added and mixed 1 µL of fungicide solution, and formed a pyramid-shaped pollen provision with a 1 mm deep hole where the egg was placed using a mini-spatula (Supplementary Fig. 1c,d). Falcon plates were held at room temperature (25 °C) and 70% RH^19^. We compared them against control larvae fed on homogenized pollen provisions treated with pure water. We recorded mortality and measured larval weight every other day until the prepupal larval instar using an analytical scale (Fisher Scientific, accuracy = 0.0001 g). Finally, the sex ratio was evaluated by dissecting cocoons after 2.5 months.

**Table 1:** Fungicide treatments.

Active ingredient	Formulation	Rate/A (100 gal)	Grams ai/100 gal	Rate formulation/L	ppm
Myclobutanil	1.67 lb ai/gal	4.8 fl oz	28.44 g	0.375 ml	75.12
	136.08 g	142.08 ml			
Penthiopyrad	1.67 lb ai/gal	16 fl oz	94.69 g	1.251 ml	250.14
trifloxystrobin	4.05 lb ai/gal	2.9 fl oz		0.227 ml	110.06
Cyprodinil	75 WDG	5 oz	106.31 g	0.375 g	280.85
Captan	80WDG	3 lb	1088.64 g	2.876 g	2875.88
Mancozeb manganese-zinc ethylene bis (dithiocarbamate)	75WDG	3 lb	1020.60 g	2.876 g	2696.14

### Metagenome sample preparation and sequencing

DNA was extracted from whole *O. cornifrons* larvae (*n* = 3 per treatment condition, mancozeb-treated and untreated pollen provisions), we conducted a microbial diversity analysis on these samples, particularly because the highest mortality rates were observed in larvae fed on mancozeb-treated pollen provisions.. Using the DNAZymoBIOMICS^®^-96 MagBead DNA Kit (Zymo Research, Irvine, CA), DNA was amplified, purified, and sequenced on Illumina^®^ MiSeq™ with a v3 reagent kit (600 cycles). Bacterial 16S ribosomal RNA gene-targeted sequencing was performed using the Quick-16S™ NGS Library Prep Kit (Zymo Research, Irvine, CA), employing primers that target the V3-V4 region of the 16S rRNA gene. Additionally, 18S sequencing was conducted with 10% PhiX spike-in, and amplification was performed using the primer pair 18S001 and NS4.

### Taxonomic profiling

Paired-end reads were imported and processed using the QIIME2 (v2022.11.1) pipeline^39^. These reads were trimmed and merged, and chimeric sequences were removed using the DADA2 plugin in QIIME2 (qiime dada2 denoise-paired)^40^. Taxonomic assignment for 16S and 18S was performed with the feature-classifier classify-sklearn plugin and the pre-trained silva-138-99-nb-classifier artifact.

### Statistical analysis

All experiments' data were tested for normality (Shapiro-Wilks) and homogeneity of variance (Levene’s test) assumptions. Since data sets did not meet the assumptions for parametric analysis and transformation failed to normalize the residuals, we employed nonparametric two-way ANOVAs (Kruskal–Wallis) with two factors [time (three time points 2, 5 and 8 days) and fungicide] to assess the effect of treatments on larval fresh weight, then posthoc nonparametric pairwise comparisons were conducted using Wilcoxon test. We used a generalized linear model (GLM) with a Poisson distribution to compare the impacts of fungicides on survival at three fungicide concentrations^41,42^. For differential abundance analysis, amplicon sequence variant counts (ASVs) were collapsed at the genus level. Differential abundance comparisons between groups using 16S (genus level) and 18S relative abundances were performed using a Generalized Additive Model for Location, Scale, and Shape (GAMLSS) with zero-inflated beta (BEZI) family distribution, implemented in the metamicrobiomeR^43^ (v1.1). Mitochondria and Chloroplast genera were removed before differential analysis. Due to the differing levels of the 18S classification, only the lowest level of each taxon was used for differential analysis. All statistical analyses were conducted using R (v. 3.4.3., CRAN project) (Team 2013).

## Results

### Larval weight gain

**Figure 1:**
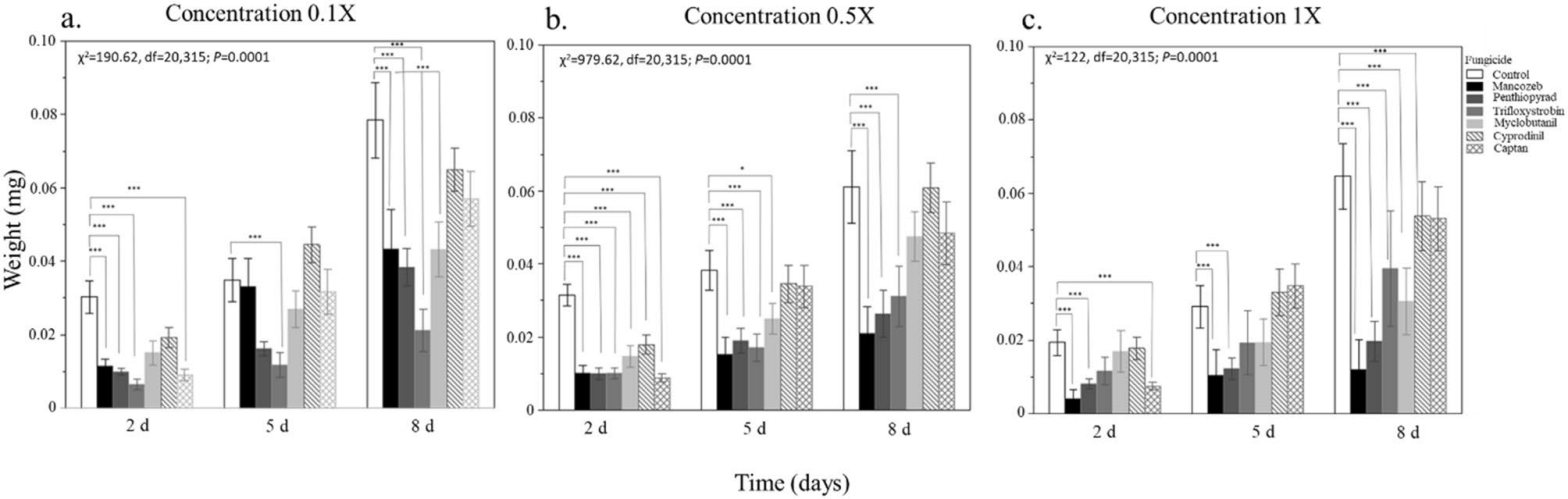
Mean fresh weights of *Osmia cornifrons* larvae measured at three-time points across four diet treatments (homogenized pollen provisions + fungicides: control, 0.1X, 0.5X, and 1X dosage. (**a**) Low dose (0.1X): First time point (1st day): χ^2^:30.99, DF = 6; *P* < 0.0001, second time point (5th day): 22.83, DF = 6; *P* = 0.0009; third time point (8th day): χ^2^:28.39, DF = 6; *P* < 0.0001. (**b**) Half dose (0.5X): First time point (1st day): χ^2^:35.67, DF = 6; *P* < 0.0001, second time point (1st day): χ^2^:15.98, DF = 6; *P* = 0.0090; third time point (8th day) χ^2^:16.47, DF = 6; *P* = 0.0041. (**c**) Field or full dose (1X):First time point (1st day) χ^2^:20.64, DF = 6; *P* = 0.0326, second time point (5th day): χ^2^:22.83, DF = 6; *P* = 0.0009; third time point (8th day): χ^2^:28.39, DF = 6; *P* < 0.0001. Two-way nonparametric anovas, followed by pairwise comparisons (α = 0.05) (*n* = 16). Bars represent mean ± S.E. *P ≤ 0.05, **P ≤ 0.001, ***P ≤ 0.0001.

Exposure to mancozeb, penthiopyrad, and trifloxystrobin significantly reduced the weight gain in *O. cornifrons* (Fig. 1)*.* These effects were consistently observed in the three evaluated doses (Fig. 1a–c). Cyprodinil and myclobutanil did not significantly reduce the larval weight.

At the lowest dosage (0.1X), larval weight exhibited a reduction of 60% with trifloxystrobin exposure, 49% with mancozeb, 48% with myclobutanil, and 46% with penthiopyrad (Fig. 1a). When exposed to half of the field dose (0.5X), larval weight decreased by 86% with mancozeb, 52% with penthiopyrad, and 50% with trifloxystrobin (Fig. 1b). Full field dose (1X) reduced larval weight in an 82% with mancozeb, 70% with penthiopyrad, and approximately 30% with trifloxystrobin, myclobutanil, and sangard (Fig. 1c).

### Mortality

**Figure 2:**
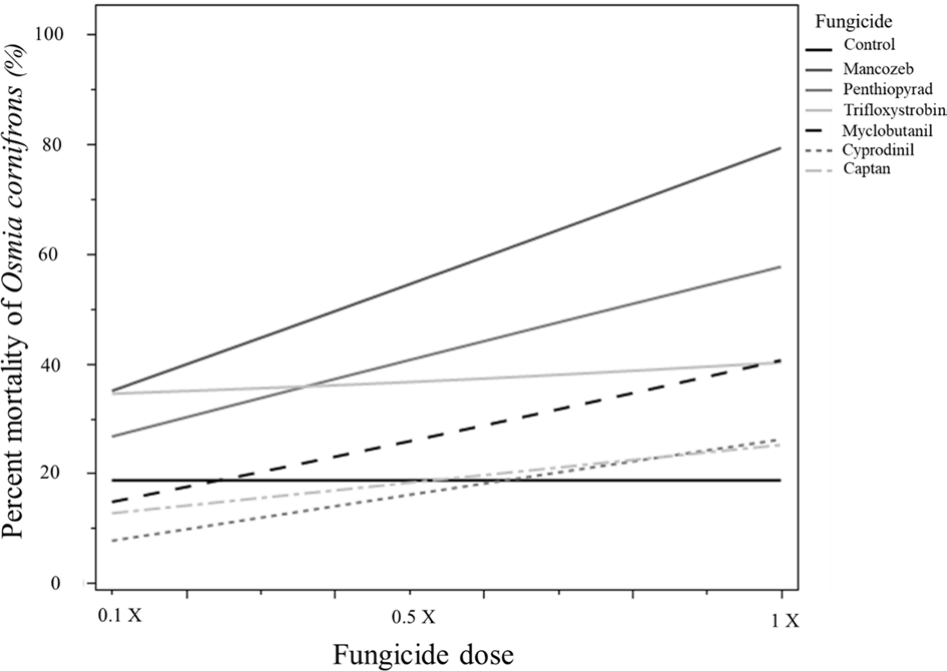
Comparison of *Osmia cornifrons* larvae mortality after ingestion of pollen provision individually treated with six different fungicides. Susceptibility of *O. cornifrons* larvae by oral exposure was high with mancozeb and penthyopirad (GLM: χ^2^ = 29.45, DF = 20, *P* = 0.0059) (line, slope = 0.29, *P* < 0.001; slope = 0.24, *P* < 0.00, respectively).

The highest mortality was observed in larvae fed on pollen treated with mancozeb, followed by penthiopyrad and trifloxystrobin. Mortality increased with the dose of mancozeb and penthiopyrad (Fig. 2; Table 2). However, *O. cornifrons* mortality only slightly increased as the concentration of trifloxystrobin rose; cyprodinil and captan did not significantly increase mortality compared to the control treatment.

**Table 2:** Estimated effects of fungicides on the mortality of *Osmia cornifrons*, AICc = 465.10.

Term	Estimate	Std error	L-R ChiSquare	Prob > ChiSq	Lower CL	Upper CL
Intercept	− 1.323524	0.1163057	234.34666	< .0001*	− 1.566385	− 1.10677
Fungicide 2[Captan]	− 0.389714	0.3154455	1.7272449	0.1888	− 1.083876	0.1762354
Fungicide 2[Control 2]	− 0.350453	0.304782	1.4622767	0.2266	− 1.007878	0.2019579
Fungicide 2[Flint]	0.3385535	0.2499376	1.687016	0.1940	− 0.184721	0.8042187
Fungicide 2[Fontelis]	0.3940779	0.2313536	2.6284852	0.1050	− 0.08813	0.8283621
Fungicide 2[Manzate]	0.6963183	0.2068337	9.8458104	0.0017*	0.2745076	1.091384
Fungicide 2[Sonoma]	− 0.06802	0.2788816	0.0607018	0.8054	− 0.66793	0.4410424
Concentration[1/2x]	− 0.016317	0.1605616	0.0103472	0.9190	− 0.339716	0.2955411
Concentration[1/10x]	− 0.326182	0.1795397	3.6978471	0.0545	− 0.714821	0.0059858
Concentration[1/2x]*Fungicide 2[Captan]	0.0555777	0.4371772	0.0160488	0.8992	− 0.881034	0.8995513
Concentration[1/2x]*Fungicide 2[Control 2]	0.0163167	0.4295465	0.0014394	0.9697	− 0.911635	0.830548
Concentration[1/2x]*Fungicide 2[Flint]	− 0.161864	0.3497726	0.2177642	0.6407	− 0.888416	0.5084733
Concentration[1/2x]*Fungicide 2[Fontelis]	0.1190838	0.3158973	0.1409388	0.7073	− 0.520681	0.7347407
Concentration[1/2x]*Fungicide 2[Manzate]	− 0.049625	0.2911959	0.0291654	0.8644	− 0.641128	0.5123676
Concentration[1/2x]*Fungicide 2[Sonoma]	− 0.266116	0.4115738	0.4389015	0.5077	− 1.170282	0.4989157
Concentration[1/10x]*Fungicide 2[Captan]	− 0.040023	0.4870944	0.0068173	0.9342	− 1.165051	0.8434346
Concentration[1/10x]*Fungicide 2[Control 2]	0.3261815	0.436995	0.5295615	0.4668	− 0.611501	1.1602425
Concentration[1/10x]*Fungicide 2[Flint]	0.3303224	0.3476476	0.8858174	0.3466	− 0.371011	1.014296
Concentration[1/10x]*Fungicide 2[Fontelis]	− 0.130667	0.3629818	0.1326051	0.7157	− 0.908785	0.5496485
Concentration[1/10x]*Fungicide 2[Manzate]	− 0.027442	0.3180726	0.0074661	0.9311	− 0.683656	0.5847395
Concentration[1/10x]*Fungicide 2[Sonoma]	0.0437489	0.4193417	0.0107985	0.9172	− 0.870253	0.8307765

### Sex ratio changes

**Figure 3:**
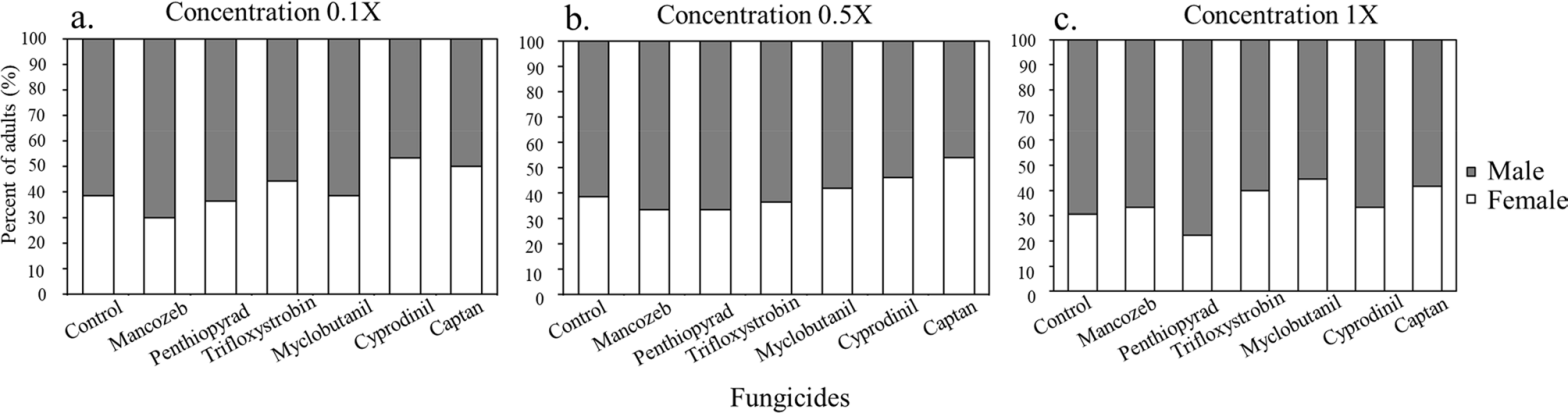
Percent of females and males of *Osmia cornifrons* after exposure to fungicides during larval stage. (**a**) Low dose (0.1X). (**b**) Half dose (0.5X). (**c**) Field or full dose (1X).

On average, 39.05% of individuals were females, while 60.95% were males across all treatments. In the control treatment, the proportion of females was observed to be 40% in both the low dose (0.1X) and half dose (0.5X) trials, and 30% in the field dose trial (1X). At the 0.1X dose, 33.33% of adults were females in larvae fed on pollen treated with mancozeb and myclobutanil, 22% for penthiopyrad, 44% for myclobutanil,41% for captan, and 31% for the control (Fig. 3a). At 0.5X dose, 33% of adults were females in mancozeb and penthiopyrad, 36% for trifloxystrobin, 41% for myclobutanil, 46% for cyprodinil, 53% for captan, and 38% for the control (Fig. 3b). At 1X dose, 30% were females for mancozeb, 36% for penthiopyrad, 44% for trifloxystrobin, 38% for myclobutanil, 50% for cyprodinil, and 38.5% for the control (Fig. 3c).

### Microbiome

**Figure 4:**
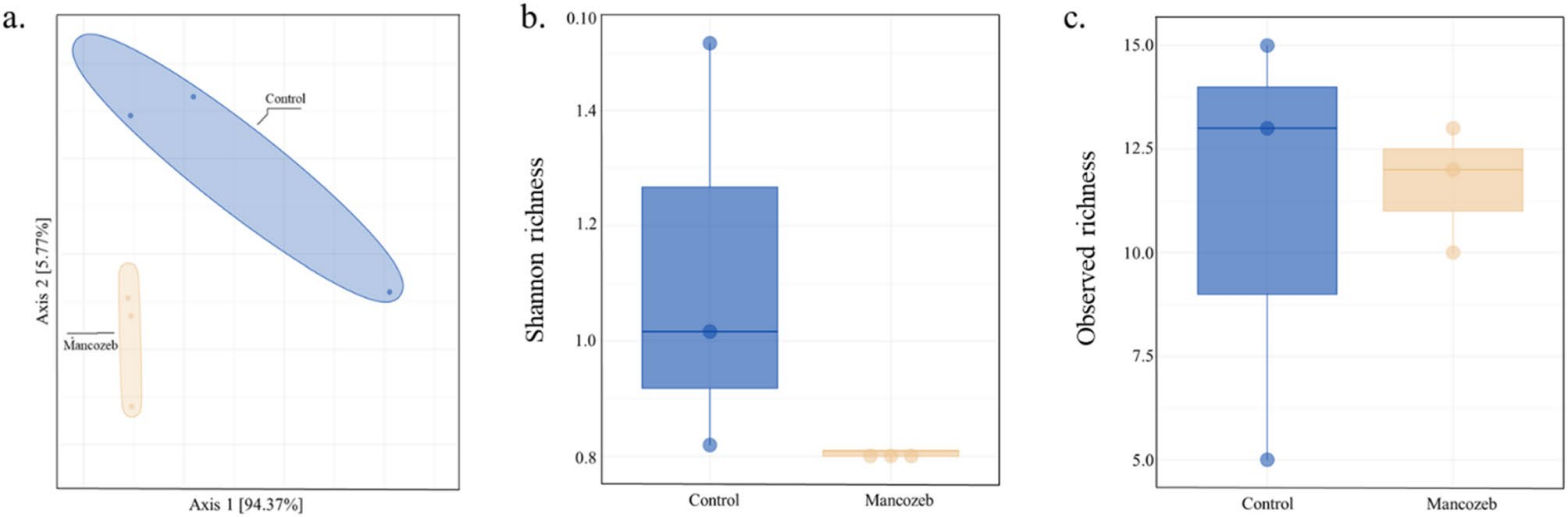
Comparisons of the Shannon richness and observed richness at the phylum level based on SAV profile for 16S sequences. (**a**) Principal coordinate analysis (PCoA) based on the overall structure of microbial communities in untreated pollen-fed larvae or control (blue) and mancozeb-fed larvae (orange). Each data point represents an individual sample. PCoA was calculated using Bray–Curtis distances with a multivariate t-distribution. Ellipses represent an 80% confidence level. (**b**) Boxplots, raw data (points) of Shannon richness, and c. observed richness. Box plots display the median line, interquartile range (IQR) boxes, and 1.5 × IQR (*n* = 3).

**Figure 5:**
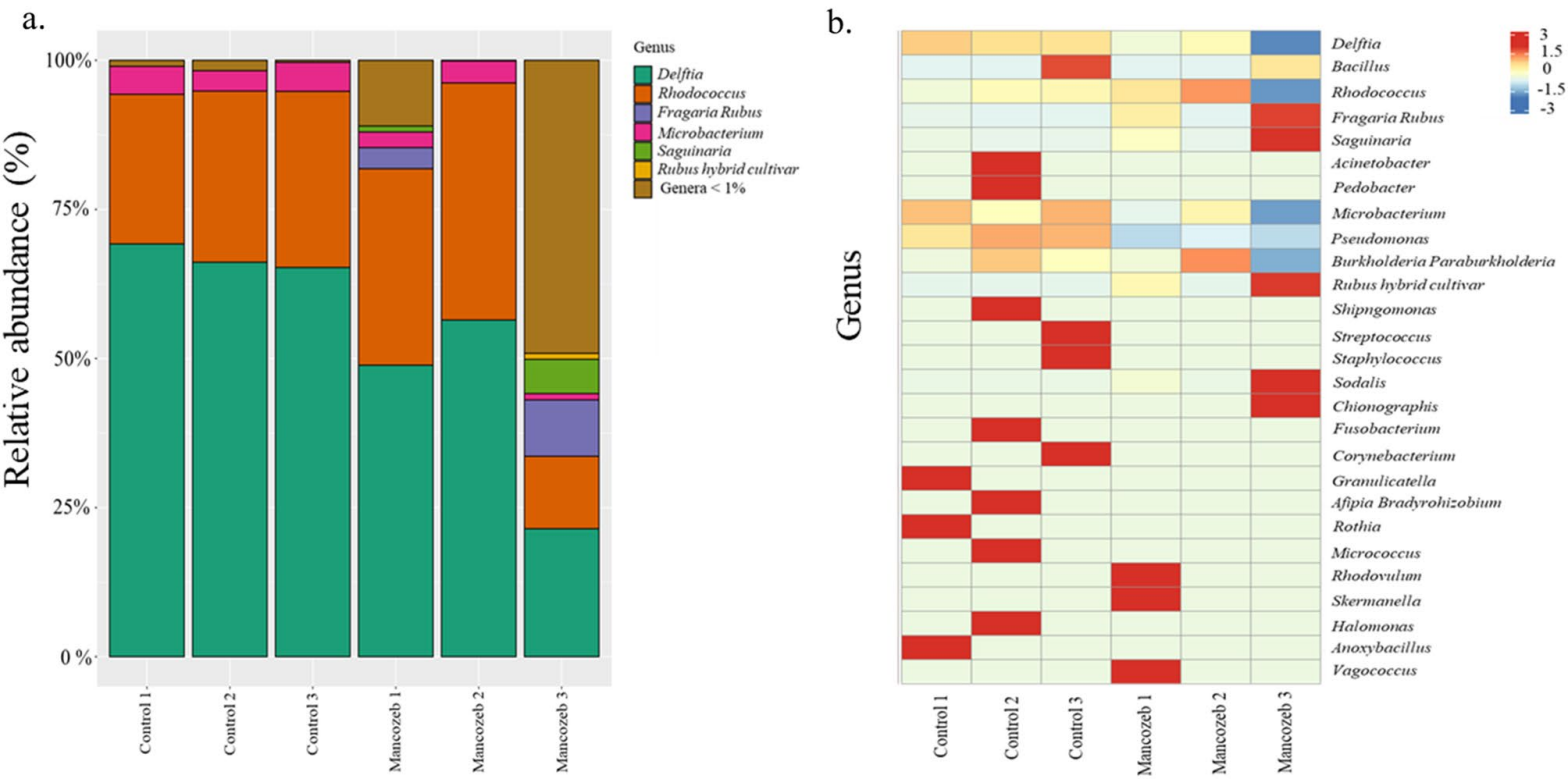
Composition of microbial communities in larva fed on mancozeb-treated and untreated pollen provisions. (**a**) Relative abundance of reads of microbial genera in larvae. (**b**) Heatmap of microbial communities identified. *Delftia* (odds ratio (OR) = 0.67, *P* = 0.0030) and *Pseudomonas* (OR = 0.3, *P* = 0.0074), *Microbacterium* (OR = 0.75, *P* = 0.0617); *Rhodococcus* (OR = 1.5, *P* = 0.0060). Heatmap rows are clustered using correlation distance and average linkage.

Analysis of 16S sequences showed that the bacteriome differed between larvae fed on mancozeb-treated pollen and those fed on untreated pollen (Fig. 4a). The microbial index was higher in untreated pollen-fed larvae than larvae fed on mancozeb-treated pollen (Fig. 4b). While the difference in observed richness between the groups was not statistically significant, it was markedly lower than in larvae fed on untreated pollen (Fig. 4c). The relative abundance showed that the microbiome of larvae fed on control pollen was more diverse than in those fed on mancozeb-treated (Fig. 5a). Descriptive analysis indicated the presence of 28 genera across the control and mancozeb-treated samples (Fig. 5b). c Analysis using 18S sequencing did not exhibit significant differences (Supplementary Fig. 2).

## Discussion

Our results suggest that oral exposure to contact (mancozeb) and systemic (penthiopyrad and trifloxystrobin) fungicides which are widely applied during bloom significantly reduced weight gain and increased mortality in *O. cornifrons* larvae. Additionally, mancozeb notably reduced the microbiome's diversity and richness at the pre-pupal stage. Another systemic fungicide, myclobutanil, significantly reduced larval weight gain at all three doses. This effect was notable at the second (5th day) and third (8th day) time points. In contrast, cyprodinil and captan did not significantly reduced weight gain or survival compared to the control group. To our knowledge, this work is the first to determine the effects of field doses of a wide range of fungicides used in crop protection on *O. cornifrons* using direct exposure through pollen provisions.

All fungicide treatments significantly reduced weight gain compared to control treatments. Mancozeb had the most substantial impact on larval weight gain, declining 51% on average, followed by penthiopyrad. However, other studies did not report the adverse effects of a fungicide field dose on the larval stage^44^. Although dithiocarbamate fungicides have been shown to have low acute toxicity^45^, Ethylenebisdithiocarbamate (EBDCS), such as mancozeb, can degrade into ethylene thiourea. Given its reported mutagenic impacts on other animals, this degradation product may be responsible for the observed effects^46,47^. Previous research indicated that the formation of ethylene thiourea is influenced by factors such as increasing temperature^48^, moisture levels^49^, and length of product storage period^50^. Proper storage conditions for the fungicide could mitigate these adverse effects. In addition, concern about the toxicity of penthiopyrad has been raised by the European Food Safety Authority, which found carcinogenic effects in the digestive system of other animals^51^.

Oral ingestion of mancozeb, penthiopyrad, and trifloxystrobin increased the mortality of *O. cornifrons* larvae. In contrast, myclobutanil, cyprodinil, and captan did not affect mortality. These results diverge from Ladurner et al.^52^, which showed captan significantly reduced survival in *O. lignaria* and *Apis mellifera* L. (Hymenoptera, Apidae) adults. Furthermore, fungicides, such as captan and boscalid, were found to induce larval mortality^52–54^ or altered foraging^55^. These changes could, in turn, affect the nutritional quality of pollen, ultimately impacting the energy gain during the larval stage. The mortality observed in control groups was consistent with those reported in other studies^56,57^.

The male-biased sex ratio observed in our work can be attributed to factors such as insufficient mating^58^ and adverse weather conditions during the blooming period^59^, as previously suggested by Vicens & Bosch^60^ for *O. cornuta.* Although females and males in our study were given four days for mating—a period generally considered adequate for successful mating^38^—we intentionally reduced the light intensity to minimize stress. The modification, however, may have inadvertently hindered the mating process^61^. Furthermore, the bees were exposed to several days of unfavorable weather, including rain and cold temperatures (< 5 °C), which could have also negatively impacted mating success^4,23^.

Although our study focuses on the microbiomes of whole larvae, our findings provide insights into potential relationships among bacterial communities that may be crucial for bee nutrition and fungicide exposure. For instance, larvae fed on pollen treated with mancozeb exhibited a significant reduction in both the structure and abundance of their microbial communities compared to those fed on untreated pollen. In larvae that consumed untreated pollen, the dominant bacterial groups were Proteobacteria and Actinobacteria, which are primarily aerobic or facultatively aerobic. *Delftia* bacteria, often associated with solitary bee^62^ species, are known to exhibit antibiotic activity, suggesting their potential protective role against pathogens^62–64^. Another bacterial genus that was abundant in larvae fed on untreated pollen but significantly reduced in mancozeb-treated larvae was *Pseudomonas*. Our results corroborate previous studies that identified *Pseudomonas* as one of the most abundant genera in *O. bicornis*^35^ and other solitary bees^34^. Although experimental evidence of the *Pseudomonas’* role in *O. cornifrons* health has not been studied, this bacterium has been shown to contribute to the synthesis of a defensive toxin in the beetle, *Paederus fuscipes*, and promotes arginine metabolism under in vitro conditions^35,65^. These observations suggest its potential role in viral and bacterial defense during *O. cornifrons* larval development. *Microbacterium* was another genus detected in our study, which has been reported in high abundance in black soldier fly larvae under starvation field conditions^66^. In *O. cornifrons* larvae, *Microbacterium* could contribute to the balance and resiliency of the gut microbiome under stress conditions. In addition, *Rhodococcus* was found in *O. cornifrons* larvae and is known for its detoxification capabilities^67^. This genus has also been found in the guts of *A. florea* but at very low abundance^68^. Our results indicate a diverse range of genetic variations across numerous microbial taxa that may alter metabolic processes in larvae. However, the functional diversity of bacteria in *O. cornifrons* needs to be better understood.

To conclude, the results suggest that mancozeb, penthiopyrad, and trifloxystrobin reduced weight gain and increased mortality of *O. cornifrons* larvae. Although there is increasing interest in the impacts of fungicides on pollinators, the effects of residual metabolites of these compounds need to be better understood. These results may be incorporated into recommendations for integrated pollinator management programs, which can instruct farmers to avoid certain fungicides during the prebloom and bloom period of fruit trees by fungicide selection and application timing change or promot use of less detrimental alternatives^36^. This information has critical implications for establishing pesticide application recommedations such as adjusting the existing spray program in fungicide selection and spray timing change or promote use of less detrimental alternatives. Additional studies are needed on the side effects of fungicides on sex ratio, foraging behavior, gut microbiome, and the molecular mechanisms underlying *O. cornifrons* weight loss and mortality.

### Supplementary Information

Supplementary Information.

## Data Availability

The source data underlying Figs. 1, 2 and 3 has been deposited in the figshare data repository DOI: 10.6084/m9.figshare.24996245 and 10.6084/m9.figshare.24996233. The sequences analysed during the current study (Figs. 4, 5) are available in the NCBI SRA repository, Accession PRJNA1023565.
