# Author Correction: Fungicide ingestion reduces net energy gain and microbiome diversity of the solitary mason bee

**DOI:** 10.1038/s41598-024-56482-8

**Published:** 2024-03-18

**Authors:** Mitzy F. Porras, Juan Antonio Raygoza Garay, Malachi Brought, Tomas López–Londoño, Alexander Chautá, Makaylee Crone, Edwin G. Rajotte, Ngoc Phan, Neelendra K. Joshi, Kari Peter, David Biddinger

**Affiliations:** 1 Department of Entomology, The Pennsylvania State University, 501 ASI Bldg, University Park, USA; 2 Department of Communication Sciences and Disorders, Holden Comprehensive Cancer Center, University of Iowa, 200 Hawkins Dr, Iowa City, IA 52242 USA; 3 Department of Biology, The Pennsylvania State University, 208 Mueller Lab, University Park, PA16802 USA; 4 Department of Ecology, Cornell University, Ithaca, NY 14850 USA; 5 Center for Pollinator Research, Intercollege Graduate Program in Ecology, Huck Institutes of the Life Sciences, Pennsylvania State University, University Park, PA16802 USA; 6 Department of Entomology and Plant Pathology, University of Arkansas, Fayetteville, AR 72701 USA; 7 Department of Plant Pathology and Environmental Microbiology, Fruit Research and Extension Center, Pennsylvania State University, 290 University Dr., Biglerville, PA 17307 USA; 8 Department of Entomology, Fruit Research and Extension Center, 290 University Dr., Biglerville, PA 17307 USA; 9 Present Address: Department of Biology, San Francisco State University, 1600 Holloway Avenue, San Francisco, CA 94132 USA

Correction to: *Scientific Reports* 10.1038/s41598-024-53935-y, published online 08 February 2024

The original version of this Article contained an error in the name of author Neelendra K. Joshi, which was incorrectly given as Neelandra K. Joshi.

The original Article and accompanying Supplementary Information file have been corrected.

