# Supplementary material for: Fungicide ingestion reduces net energy gain and microbiome diversity of the solitary mason bee
Source: Sci Rep. 2024 Feb 8;14:3229. doi: 10.1038/s41598-024-53935-y (PMC10853529; doi:10.1038/s41598-024-53935-y)
Supplement: Supplementary file 1 — Supplementary Information. [file 41598_2024_53935_MOESM1_ESM.docx]

Ingestion of fungicides reduces net energy gain and microbiome diversity of the solitary mason bee

Mitzy F. Porras ^1*†^, Juan Antonio Raygoza Garay ^2^, Malachi Brought ^1^, Tomas López–Londoño ^3^, Alexander Chautá ^4^, Makaylee Crone ^5^, Edwin G. Rajotte ^1^, Ngoc Phan ^6^, Neelendra Joshi ^6^, Kari Peter ^7^, David Biddinger ^8^

^1^ Department of Entomology, 501 ASI Bldg, The Pennsylvania State University, University Park, ^†^ Department of Biology, San Francisco State University. 1600 Holloway Avenue, San Francisco, CA 94132 USA

^2^ Holden Comprehensive Cancer Center, Dept. of Communication Sciences and Disorders University of Iowa, 200 Hawkins Dr, Iowa City, IA 52242 USA

^3^ Department of Biology, The Pennsylvania State University, 208 Mueller Lab, University Park, PA16802, USA

^4^ Department of Ecology, Cornell University, Ithaca, NY 14850 USA

^5^ Intercollege Graduate Program in Ecology, Center for Pollinator Research, Huck Institutes of the Life Sciences, Pennsylvania State University, University Park, PA16802, USA

^6^ Department of Entomology and Plant Pathology, University of Arkansas, Fayetteville, AR 72701, USA

^7^ Department of Plant Pathology and Environmental Microbiology, Fruit Research and Extension Center, Pennsylvania State University, 290 University Dr., Biglerville, PA 17307 USA

^8^ Department of Entomology, Fruit Research and Extension Center.290 University Dr., Biglerville, PA 17307 USA

^†^ Current address

*Corresponding author: Mitzy Porras, E-mail: mitzy.porras@gmail.com, +1 765 409 2795.

| **Table S1.** Fungicides used in bioassays, commercial name, common name, and mode of action group for each pesticide are given. *FRAC: Fungicide Resistance Action Committee. |
| --- |
| \| **Product** \| **Formulation** \| **Chemical Group**  **(FRAC Code)*** \| \| --- \| --- \| --- \| \| \| Captan 80WDG  *(United Phosphorus, Inc.,*  *King of Prussia, PA)* \| Captan 80% \| M4 – phthalimides \| \| Flint Extra 4.05F  *(Bayer Crop Sciences LP,*  *St. Louis, MO)* \| Trifloxystrobin 42.6% \| 11 – Quinone Outside Inhibitors (Qol) \| \| Fontelis 1.67F  *(Corteva Agriscience,*  *Indianapolis, IN)* \| Penthiopyrad 20.4% \| 7 – Succinate- Dehydrogenase Inhibitors (SDHI) \| \| Manzate Pro-Stick 75DG  *(United Phosphorus, Inc.,*  *King of Prussia, PA)* \| Mancozeb 75% \| M3- Dithiocarbamates \| \| Sonoma 20EW AG  *(Albaugh LLC,*  *Ankeny, IA)* \| Myclobutinol 19.7% \| 3 - DeMethylation Inhibitors DMI) \| \| Vangard WG \| Cyprodinil 75% \| 9 - Anilino- Pyrimidines (AP \| |

**Supplementary Figure 1**

**
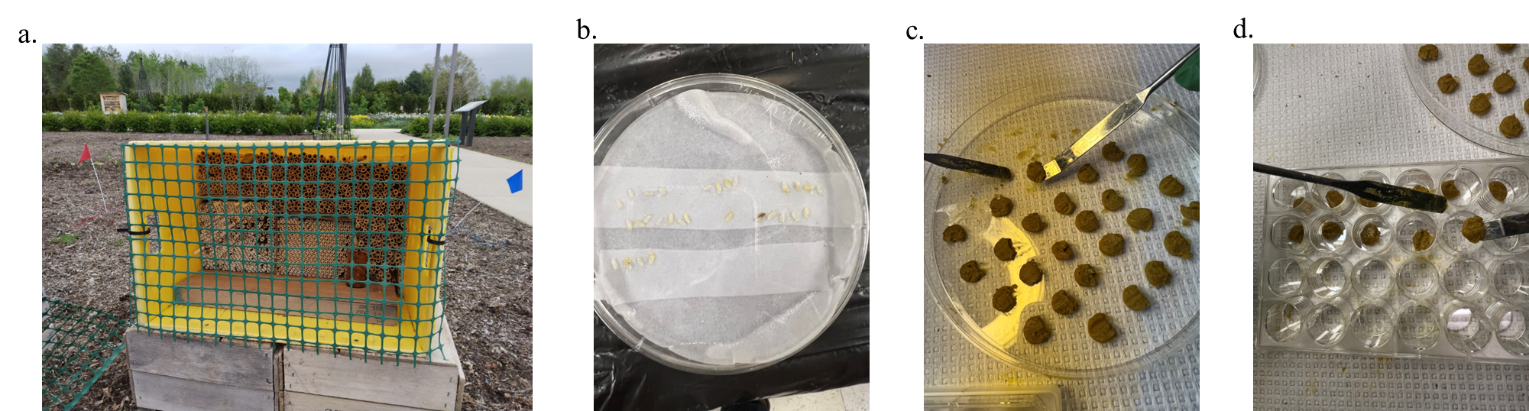
**

**Figure S1**. Experimental set up to obtain *Osmia cornifrons* eggs. a. Nest trap located at the Arboretum, Penn State main campus, boxes were sprayed with nesting pheromone (InvitaBee™Plus+) supplied by Dr. Theresa Pitts Singer and Natalie Boyle to ensure that mason bees return to their hives. b. Egg incubation. c-d homogenized pollen provisions.

**Supplementary Figure 2**


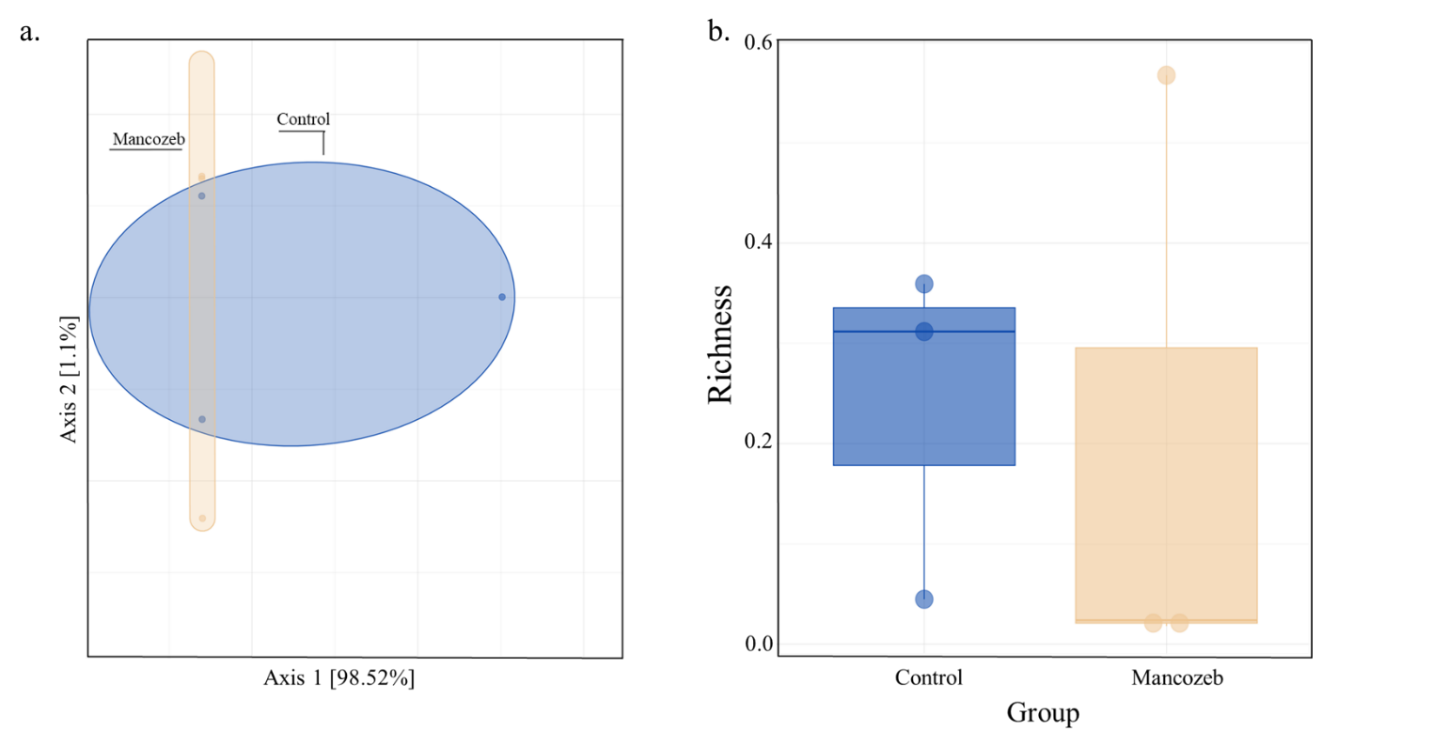


**Figure 2**. Comparisons of the Shannon richness and observed richness at the phylum level based on SAV profile 18S. a. Principal coordinate analysis (PCoA) based on the overall structure of microbial communities in untreated pollen-fed larvae or control (blue) and mancozeb-fed larvae (orange). Each data point represents an individual sample. PCoA was calculated using Bray-Curtis distances with a multivariate t-distribution. Ellipses represent an 80% confidence level. b. Boxplots, raw data (points) of Shannon richness, and c. observed richness. Box plots display the median line, interquartile range (IQR) boxes, and 1.5 x IQR (*n* = 3).
